# Supplementary material for: Synergistic Arg-C Ultra and Lys-C Digestion for Quantitative Proteomics
Source: bioRxiv. 2025 Jul 18:2025.07.15.664461. Preprint. [Version 1] doi: 10.1101/2025.07.15.664461 (PMC12338676; doi:10.1101/2025.07.15.664461)
Supplement: 1 [file NIHPP2025.07.15.664461v1-supplement-1.pdf]

bioRxiv preprint doi: https://doi.org/10.1101/2025.07.15.664461; this version posted July 18, 2025. The copyright holder for this preprint (which was not certified by peer review) is the author/funder, who has granted bioRxiv a license to display the preprint in perpetuity. It is made available under aCC-BY 4.0 International license.

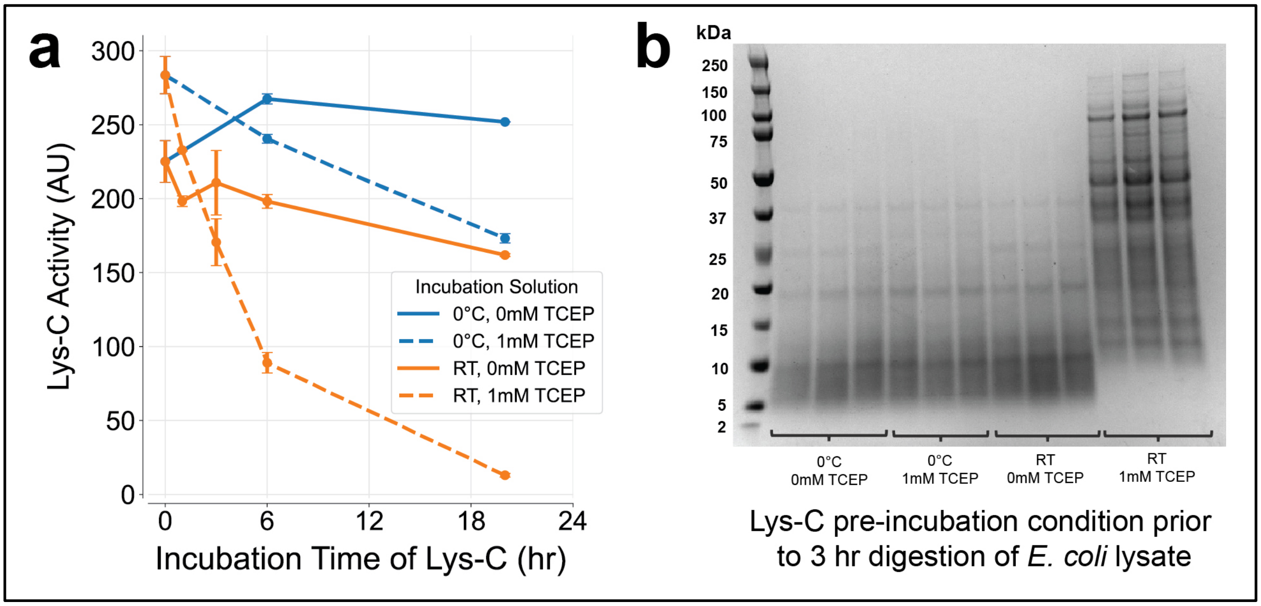

Figure S1. Validation that 1 mM TCEP Inhibits Lys-C Activity.

(a) TCEP exposure strongly reduces Lys-C activity, measured via UV-Vis. Lys-C was incubated in 20 mM EPPS pH 8.5 with or without 1 mM TCEP at the indicated temperatures. Activity over time was measured using UV–Vis as in Figure 4. Exposure to TCEP at room temperature led to a >10-fold reduction in enzymatic activity after 20 hours. Error bars represent ±1 standard deviation from n=3 replicates.

(b) Pre-incubation with 1mM TCEP renders Lys-C unable to digest lysate. Lys-C was pre-incubated in the indicated solutions overnight, then added to precipitated *E. coli* lysate for a 3-hour digestion at room temperature. The supernatant was collected, and proteins still bound to the beads were additionally released. Coomassie staining shows that Lys-C fails to digest the lysate when pre-incubated with 1 mM TCEP at room temperature.

bioRxiv preprint doi: https://doi.org/10.1101/2025.07.15.664461; this version posted July 18, 2025. The copyright holder for this preprint (which was not certified by peer review) is the author/funder, who has granted bioRxiv a license to display the preprint in perpetuity. It is made available under aCC-BY 4.0 International license.

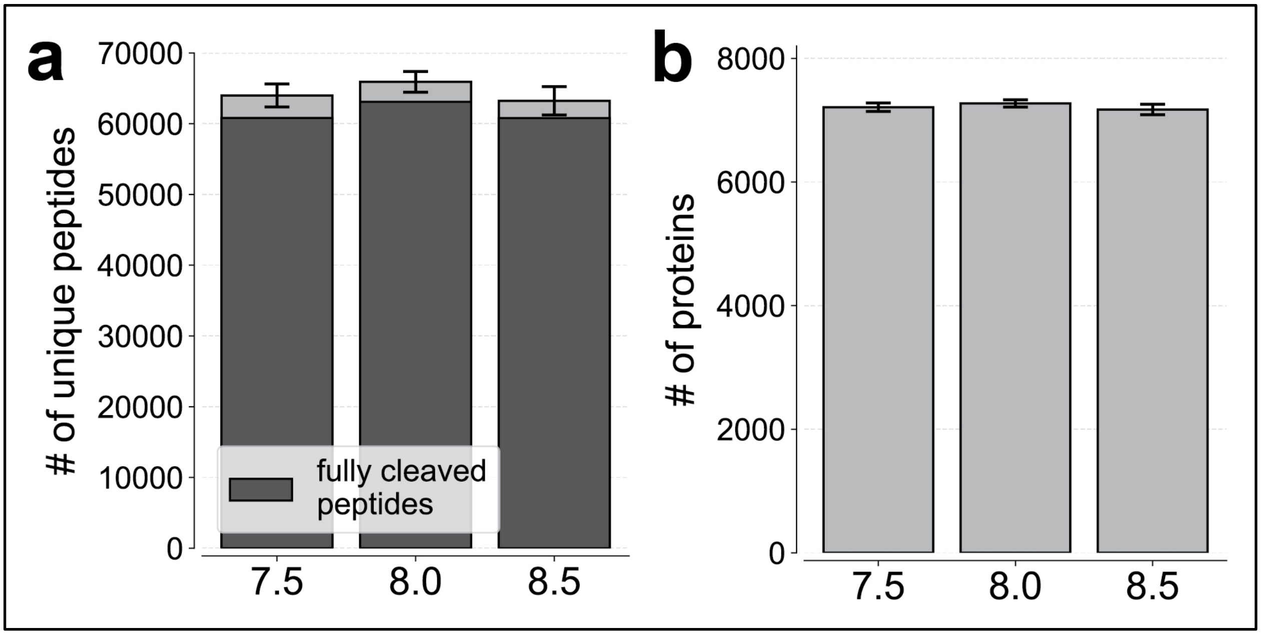

Figure S2. Effect of pH on digestion with the sequential Lys-C/Arg-C Ultra workflow.
(a) Number of unique peptides. Bead-bound proteins were digested overnight with Lys-C (40 ng/uL) in 20 mM EPPS at pH 7.5, 8.0, or 8.5 (37 °C). TCEP (1 mM) and Arg-C Ultra (1:200 enzyme-to-protein) were then added for a second overnight digestion at 24 °C. All pH conditions performed similarly. Error bars represent ±1 standard deviation from n=3 replicates (digested independently). Data were acquired by label-free DIA on an Orbitrap Ascend.
(b) Number of proteins. All pH conditions performed similarly
